# Explaining variation in adolescents’ social media-related distraction: The role of social connectivity and disconnectivity factors

**DOI:** 10.1007/s12144-022-03844-y

**Published:** 2022-11-24

**Authors:** Teun Siebers, Ine Beyens, J. Loes Pouwels, Patti M. Valkenburg

**Affiliations:** 1 Amsterdam School of Communication Research, University of Amsterdam, P.O. Box 15791, 1001 NG Amsterdam, The Netherlands; 2 Behavioural Science Institute, Radboud University, Nijmegen, The Netherlands

**Keywords:** ESM, Instagram, Attention, *N* = 1 research, Media effects

## Abstract

Social media are often believed to distract adolescents’ attention. While existing research has shown that some adolescents experience more social media-related distraction than others, the explanations for these differences remain largely unknown. Based on Self-Determination Theory, this preregistered study investigated two social connectivity factors (fear of missing out [FoMO] and friendship accessibility expectations) and two disconnectivity factors (self-control strategies and parental restrictions) that may explain heterogeneity in social media-related distraction. We used data collected through a measurement burst design, consisting of a three-week experience sampling method study among 300 adolescents (21,970 assessments) and online surveys. Using *N* = 1 analyses, we found that most adolescents (77%) experienced social media-related distraction. Contrary to expectations, none of the connectivity or disconnectivity factors explained differences in social media-related distraction. The findings indicate that social media are a powerful distractor many adolescents seem to struggle with.

Social media hold considerable power to distract adolescents’ attention. For instance, a majority of adolescents admit that they are often distracted by social media while doing homework or while being with others (Rideout & Robb, 2018). Moreover, both cross-sectional and longitudinal research among adolescents and young adults has shown that social media use (SMU) is related to increased distraction and related problems, such as lack of concentration (Aalbers et al., 2019; Levine et al., 2007; Siebers et al., 2021; Xie et al., 2021). Many negative consequences of social media-related distraction have been reported over the past decade, including impaired task performance (Brooks, 2015), lower academic achievement (Dontre, 2021; Rosen et al., 2013), and impaired well-being (Brooks, 2015; Johannes et al., 2020).

While growing evidence points to an association of SMU with distraction, it has recently been questioned whether such association holds for all adolescents (Siebers et al., 2021). Media effects theories, such as the Differential Susceptibility to Media Effects Model assume that the extent of social media-related distraction may differ across adolescents (Valkenburg & Peter, 2013). Nevertheless, with one exception (Siebers et al., 2021), such heterogeneity across adolescents has hardly been tested empirically. Recently, Siebers et al. found that whereas 82% of adolescents experienced social media-related distraction, 16% of adolescents experienced no change in distraction, and 2% experienced less distraction when using social media.

Although demonstrating heterogeneity in the association of SMU with distraction is a significant first step, an important gap in the literature is that we do not yet know what causes this heterogeneity. In other words, why do most adolescents experience *more* distraction at moments when they use more social media, while some others experience *less* distraction or *no* *change* in distraction at all? It is only by answering this question that we can understand which adolescents have a higher risk of experiencing attentional problems related to SMU. Such an understanding is important since maturation of attentional control, that is, the ability to focus attention and control potential distractions (Diamond, 2013), is an important developmental task for adolescents (Luna, 2009; Luna et al., 2004). In addition, only by knowing the causes of social media-related distraction, targeted interventions can be tailored to the group of adolescents who suffer most from social media-related distraction (Rodriguez et al., 2021).

The current study will address this gap in the literature by investigating why adolescents differ in the extent to which they experience social media-related distraction. Using a person-specific approach, this study investigates 1) the average within-person association of SMU with momentary distraction, 2) person-to-person heterogeneity in this association, and 3) factors that could explain person-to-person heterogeneity in this association. Based on Self-Determination Theory (SDT; Ryan & Deci, 2000), this study examines four factors that may explain heterogeneity in social media-related distraction: two social connectivity factors (i.e., fear of missing out [FoMO] and friendship accessibility expectations) and two disconnectivity factors (i.e., self-control strategies and parental restrictions). The current study uses data from a preregistered measurement burst design study that combined experience sampling method (ESM) assessments with online survey data among a sample of 300 adolescents (21,970 ESM observations in total).

## Social connectivity factors to explain heterogeneity in social media-related distraction

To understand how social connectivity factors may explain heterogeneity in social media-related distraction, we may rely on the premises of Self-Determination Theory (SDT; Ryan & Deci, 2000). Although SDT does not make any predictions on the link between social media use and distraction, it does address the mechanisms that may lead to social media-related distraction. According to SDT, adolescents have a need for relatedness or social connectivity. Social media are an important tool for adolescents to satisfy this need (Chen et al., 2021; Przybylski et al., 2013; Sheldon et al., 2011). In fact, social connectivity is one of the most important reasons why adolescents use social media (Allen et al., 2014). Adolescents’ need for social connectivity may generate social media checking routines that happen frequently, automatically, and sometimes even unnoticed (Bayer et al., 2016; Heitmayer & Lahlou, 2020). This may distract adolescents’ attention from other important tasks or goals. Therefore, the first aim of our study is to investigate the explanatory role of two connectivity factors in the association of SMU and distraction: fear of missing out (FoMO) and friendship accessibility expectations.

### Fear of missing out

Fear of missing out (FoMO) is the fear that arises when people are reminded of socially rewarding situations in which they cannot be involved (Przybylski et al., 2013). For instance, adolescents may get worried if they find out that others are having fun without them, when they miss out on a get-together, or when they do not know what others are doing. In line with the premises of SDT (Ryan & Deci, 2000), previous research suggests that adolescents whose need for social connectivity is less satisfied, experience more FoMO (Przybylski et al., 2013). Adolescents with high levels of FoMO have a strong need to constantly connect with others and a strong tendency to draw their attention to social media (Beyens et al., 2016). Research already showed that adults with higher levels of FoMO experience more social media-related distractions in a variety of contexts, for instance while studying (Al-Furaih & Al-Awidi, 2021; Przybylski et al., 2013), while interacting with others (Chotpitayasunondh & Douglas, 2016; Franchina et al., 2018; Schneider & Hitzfeld, 2021), and during daily ongoing activities (Milyavskaya et al., 2018; Rozgonjuk et al., 2019). Although these associations have not yet been investigated among adolescents, previous research suggests that FoMO is relatively stable across different age groups (Barry & Wong, 2020). Therefore, we hypothesize:

- H1: Adolescents with higher levels of FoMO experience more social media-related distraction than adolescents with lower levels of FoMO.

### Friendship accessibility expectations

Based on SDT (Ryan & Deci, 2000), it can be assumed that not only adolescents’ internal need for social connectivity may drive their SMU, but also their expectations about social connectivity towards others. The constant availability created by social media shapes expectations and demands for social connectivity, especially among adolescents (Marino et al., 2020; Nesi et al., 2018). For instance, adolescents expect that others are always accessible via social media and react quickly to their social media posts (van Driel et al., 2019). Moreover, adolescents often wait for likes or comments from others after having posted something on social media (Jong & Drummond, 2016). These friendship accessibility expectations may influence the extent to which social media distract adolescents’ attention. Specifically, adolescents who have high friendship accessibility expectations may feel a need to constantly check their social media to verify whether they have received any reactions (Bayer et al., 2016; Heitmayer & Lahlou, 2020). This heightened alertness, or online vigilance (Johannes et al., 2019), may make it difficult for these adolescents to focus and sustain their attention. Therefore, we hypothesize:

- H2: Adolescents with higher friendship accessibility expectations experience more social media-related distraction than adolescents with lower friendship accessibility expectations.

## Disconnectivity factors to explain heterogeneity in social media-related distraction

Besides a need for social connectivity, SDT argues that adolescents also have a need for autonomy and competence (Ryan & Deci, 2000). Adolescents may satisfy their need for autonomy by experiencing ownership of their behavior and decisions, and they may satisfy their need for competence by feeling efficacious and being able to successfully deal with challenges. As adolescents often use social media automatically (Bayer et al., 2016) and often experience difficulties in resisting temptations (Casey & Caudle, 2013), such as notifications and beeps, social media may frustrate adolescents’ needs for autonomy and competence. Disconnectivity (e.g., trying to avoid social media distractions) may give adolescents a sense of control, allowing them to restore their needs for competence and autonomy. Adolescents who are better able to disconnect from social media may experience fewer social media-related distractions than adolescents who are less able to do so. Therefore, the second aim of this study is to investigate the explanatory role of two disconnectivity factors in the association of SMU with distraction: self-control strategies and parental restrictions.

### Self-control strategies

Building on SDT, it can be assumed that adolescents adopt self-control strategies that prevent social media-related distractions in order to maintain a sense of competence and autonomy. Indeed, previous research pointed at a positive association between self-control and need satisfaction among adolescents in a school setting (Orkibi & Ronen, 2017). Social media self-control strategies reflect pre-determined self-imposed rules that are aimed at avoiding social media temptations that may hamper long-term goals (see Brevers & Turel, 2019; Duckworth et al., 2018). Studies have identified different strategies to avoid social media distractions (Brevers & Turel, 2019), including preventing access (e.g., making sure that the phone is not around), modifying device features (e.g., putting the phone on airplane mode), and straightforward self-control (e.g., forcing oneself not to use social media). Previous research has suggested that some adolescents exert more self-control than others (Casey & Caudle, 2013; Willems et al., 2019), which may explain why some adolescents experience more social media-related distraction than others. Based on this evidence, we hypothesize:

- H3: Adolescents who use social media self-control strategies less often experience more social media-related distraction than adolescents who use social media self-control strategies more often.

### Parental restrictions

In addition to self-control, SDT argues that adolescents’ social environment may promote their development of autonomy and competence (Ryan & Deci, 2000). According to SDT, parents may stimulate the development of autonomy and competence by engaging in autonomy-supportive parenting (Grolnick et al., 1997). Similarly, researchers have argued that parents who engage in autonomy-supportive media-specific parenting may stimulate adolescents’ autonomy development (Valkenburg et al., 2013). Such media-specific parenting may thus help adolescents to control their SMU and to prevent social media-related distraction. For instance, parents can set limits to the amount of adolescents’ screentime and restrict adolescents’ phone use at undesired moments, such as at bedtime, while doing homework, while having dinner, or while talking to someone (Shin & Li, 2016; van den Eijnden et al., 2021). However, parents differ in the extent to which they restrict adolescents’ phone use. For example, a recent study showed that about half of the parents allowed their adolescents to use their phone around bedtime, whereas the other half did not (van den Eijnden et al., 2021). Such differences in parental restrictions may explain why some adolescents experience more social media-related distraction than others. Therefore, we hypothesize:

- H4: Adolescents whose parents impose less phone restrictions experience more social media-related distraction than adolescents whose parents impose more phone restrictions.

## Methods

The current preregistered study (https://osf.io/zgr2k/) is part of a larger project that investigates the psychosocial consequences of SMU for adolescents. This larger project was approved by the Ethical Review Board of the first author’s university. The project ran from November 2019 to June 2022 and used a measurement burst design that included two three-week ESM bursts, two pre-ESM surveys, and sixteen online surveys. A previous study of this project investigated the association between SMU and distraction using data from the first ESM burst and the first pre-ESM survey (conducted in December 2019). The current study aims to extend this previous study by investigating whether the heterogeneity in the association between SMU and distraction can be explained by social connectivity and disconnectivity factors. To that end, this study used data from the second ESM burst and the second pre-ESM survey (conducted in June 2020), and four online surveys.

### Participants

Participants were recruited from a secondary school in the southern area of the Netherlands. In total, 745 adolescents were invited to participate in the larger project. A total of 400 (54%) adolescents obtained parental consent, and 388 (52%) also provided informed assent themselves to participate. A group of 312 participants participated in the second ESM burst. Twelve participants were excluded from the analyses because they did not complete the pre-ESM survey, which was necessary for receiving the ESM surveys (*n* = 8), or because they did not use social media at least once a week (*n* = 4). Thus, the final sample size consists of 300 adolescents. The mean age of participants in this sample was 14.62 (*SD* = 0.70), 58% were girls, and 96% were born in the Netherlands and identified as being Dutch. The level of education was roughly equally distributed across the sample: 37% prevocational secondary education track, 34% intermediate general secondary education track, and 29% academic preparatory education track. The sample was representative of the specific area in the Netherlands in terms of educational level and ethnic background (Statistics Netherlands, 2020).

### ESM Surveys

Shortly before the onset of the second ESM burst, adolescents received online instructions as to how to install the Ethica app that was required for receiving the ESM surveys. Upon successful installation, a pre-ESM survey was sent to participants to ask them which social media platforms they used regularly (i.e., at least once a week). During the second ESM burst, participants received six 2-min surveys each day, for 21 consecutive days (126 momentary assessments in total). The surveys were sent through the Ethica app at random time points within fixed time intervals (see https://osf.io/b8vsa/ for the trigger scheme). Participants received a notification each time when a new survey was available, and a reminder after 10 min, if needed. The number of items included in the survey varied between 19 and 32, depending on the moment of the day the survey was sent. Each survey included questions about SMU and distraction and other questions that were not used in this study. Of the 300 participants, 293 received questions about WhatsApp (98%), 261 about Instagram (87%), and 232 about Snapchat (77%). Alternative questions were used to ensure that all adolescents received the same number of questions per survey. All participants were assured that their responses would be kept anonymous and treated confidentially.

Of the 37,800 ESM surveys that were sent (300 participants * 126 momentary assessments), 21,970 were (partially) completed, resulting in a net compliance of 58%. On average, participants completed 73.23 ESM surveys (*SD* = 34.77; range = 3–126; median = 79.50). Participants received €0,30 for every completed ESM survey and €0,50 for the lengthier ESM survey at the end of the day. Moreover, participants who completed all six surveys on one day were automatically nominated for the lottery that took place on the subsequent day, in which four randomly selected participants won an additional €25. Participants were updated about their compliance and earnings on a daily basis via personal messages and via an interactive real-time monitoring website (built with Shiny R; Chang et al., 2020).

### Online surveys

From the start of the first ESM burst until the end of the second ESM burst, participants received online surveys that were accessible via Qualtrics on a biweekly basis. In total, the online surveys were distributed in sixteen waves. The variables of interest in this study were measured in wave 5 (FoMO), wave 15 (friendship accessibility expectations), and wave 14 (self-control strategies and parental restrictions). If participants had not completed the questions in the respective waves, they were offered the opportunity to complete them on a second occasion as part of the end-of-study survey in wave 16, which was administered right after the end of the second ESM burst. Participants had up to two weeks to complete each online survey. All participants were assured that their responses would be kept anonymous and treated confidentially. Completion of an online survey was rewarded with €2. Those participants who completed the online surveys within two days were automatically nominated for the lotteries in which four participants won an additional €25.

### Power analysis

The required sample size for the ESM study was based on a priori power analyses for the larger project (see https://osf.io/tk8pw/). These analyses showed that 300 participants and 63 assessments (i.e., assuming 50% compliance) were required to detect small effect sizes and variance around these effects, given 80% power and an alpha of 5%. Since the two connectivity and two disconnectivity factors were assessed in different online surveys, the actual number of participants included in the analyses depends on the number of participants who completed the social connectivity and disconnectivity items (i.e., *n* = 260 for FoMO, *n* = 256 for friendship accessibility expectations, *n* = 262 for self-control strategies, and *n* = 262 for parental restrictions). We re-estimated the preregistered power based on the smallest sample size, that is, the number of participants who completed the friendship accessibility expectation items (*n* = 256), and the average of their completed ESM surveys (i.e., 78 assessments; 19,968 in total). This resulted in 94% power to detect small effect sizes with an alpha of 5% (see https://osf.io/whnmd/), thus the smallest sample size had sufficient power to test our hypotheses.

### Measures

#### Social media use (ESM)

SMU was operationalized with three items measuring how much time in the past hour participants had spent using a specific social media platform. Based on a national survey among 14- and 15-year-olds, we selected the three most frequently used social media platforms among middle adolescents: Instagram, WhatsApp, and Snapchat (van Driel et al., 2019). For each of the social media platforms that participants used at least once a week, as indicated in the pre-ESM survey, they were asked to indicate how much time they had spent using the platform in the past hour on a visual analogue scale (VAS). The answer options ranged from 0 to 60 min, with 1-min intervals. The overall SMU variable was created by summing the three SMU items per person per measurement point. Sum scores exceeding 60 min were recoded to 60 min.

#### Distraction (ESM)

To measure distraction, participants were asked to respond to the question “To what extent were you distracted by something over the past hour?”, using a 7-point scale ranging from 0 (*not at all*) to 6 (*completely*), with 3 (*a little*) as the midpoint. This item was based on the momentary attentional control measure of Chin et al. (2020).

#### Fear of missing out (FoMO; online survey)

FoMO was measured with the five items from the Fear of Missing Out scale (FoMOs; Przybylski et al., 2013) that had the highest factor loadings within an adolescent sample (Perrone, 2016). The subset included the items 1) “I get worried when I find out that my friends are having fun without me”, 2) “I get nervous when I don't know what my friends are doing”, 3) “Sometimes I spend too much time keeping up what's going on”, 4) “When I miss out on a planned get-together it bothers me”, and 5) “It bothers me when I can't attend a meeting with friends”. Participants responded to all items on a 5-point Likert scale ranging from 1 (*totally agree*) to 5 (*totally disagree*), with 3 (*don’t agree*/*don’t disagree*) as the midpoint. The items loaded on one factor and had good reliability (Eigenvalue = 2.74; Cronbach’s α = 0.86).

#### Friendship accessibility expectations (online survey)

We measured friendship accessibility expectations using two items that we created based on insights from Hall and Baym (2012) and Nesi et al. (2018): 1) “I find it important that my close friends respond quickly via social media when I send them something” and 2) “I find it important that my close friends are always accessible via social media”. Participants responded to both items using a 5-point scale ranging from 1 (*not at all*) to 5 (*completely*), with 3 (*a little*) as the midpoint. The two items loaded on one factor and had good reliability (Eigenvalue = 1.35; Cronbach’s α = 0.81, r = 0.67).

#### Self-control strategies (online survey)

Self-control strategies were assessed using three items that were based on the Process Model of Self-Control (Duckworth et al., 2016) and on the most frequent strategies that participants used to control their SMU, which we assessed in the eleventh online survey. Participants were asked “How do you make sure social media do not distract you?” and to indicate how often they applied each of the three listed strategies: 1) “I make sure that my phone is not around”, 2) “I put my phone on silent, on airplane mode, or turn it off altogether”, and 3) “I agree with myself when I may use social media again”. Participants indicated how often they adopted the specific self-control strategy using a 5-point scale ranging from 1 (*never*) to 5 (*very often*), with 3 (*sometimes*) as the midpoint. Reliability estimates were not calculated for the items since the items represent a multidimensional rather than a unidimensional construct (Widhiarso, 2010).

#### Parental restrictions (online survey)

Parental restrictions were measured with five items that were based on the restrictive mediation subscale from the Perceived Parental Media Mediation Scale (PPMMS; Valkenburg et al., 2013). All items describe phone restrictions that may be imposed by parents: “How often do your parents tell you that you cannot use your phone…” 1) “…for too long?”, 2) “…while having dinner?”, 3) “…right before you go to sleep?”, 4) “…while doing your homework?”, 5) “…while you are talking to someone?”. Participants responded to these items using a 5-point scale ranging from 1 (*never*) to 5 (*very often*), with 3 (*sometimes*) as the midpoint. The items loaded on one factor and had good reliability (Eigenvalue = 1.78; Cronbach’s α = 0.73).

### Statistical analyses

The analyses followed our preregistered plan (see https://osf.io/srdfp). We used the statistical software Mplus version 8.6 (Muthén & Muthén, 2017) to run the analyses. To account for the hierarchical structure of the data, we adopted a multilevel modeling approach. The models were estimated using the Bayesian Markov Chain Monte Carlo (MCMC) estimation procedure, which allowed for *latent* person-mean centering. This procedure estimates latent person means (for between-person analyses) and latent person-mean centered scores (for within-person analyses) of SMU and distraction. This procedure is preferred over *observed* person-mean centering as it reduces biases and enhances interpretability (McNeish & Hamaker, 2020). An important advantage of the Bayesian approach is that it standardizes the parameters for each single person, providing *person-specific within-person standardized* parameters (Schuurman et al., 2016). An additional advantage is that it calculates uncertainty measures for each single person, including person-specific *p* values and credible intervals around the person-specific associations, to assess the significance of the effects at the *N* = 1 level (Schuurman et al., 2016).

The models were specified using a two-step model building approach. The first model (Model 1) included distraction as the outcome variable and SMU as a predictor both at the between- and within-person level (fixed effects). In addition, the model included two time-related control variables (notification number of the day and weekday/weekend day) to detrend the data (see Wang & Maxwell, 2015) and the between-person variance around the within-person association of SMU and distraction (random effect) to examine person-specific associations. In the second model (Model 2), Model 1 was extended by four cross-level interactions to examine the moderating effects of FoMO, friendship accessibility expectations, self-control strategies, and parental restrictions on the association of SMU and distraction. To do so, we correlated the scores of the moderating factors with the within-person associations of SMU with distraction. We included correlations between the moderators, SMU, and distraction to obtain more stable estimates.

Both models were estimated with 5,000 iterations and converged successfully, since the Potential Scale Reduction (PSR) values were very close to 1 (Model 1: 1.000; Model 2: 1.001), the density plots looked nice and smooth, and the trace plots looked like fat caterpillars without spikes or irregularities. We then doubled the number of iterations to rule out a potential pre-mature stoppage problem (Schultzberg & Muthén, 2018, p. 514). The PSR values for both models were 1, and the results did not deviate from the models with 5,000 iterations. The fixed effects and person-specific effects were interpreted based on the effect sizes and (Bayesian) *p* values. The smallest effect size of interest (SESOI; Lakens et al., 2018) was 0.10 for the between-person associations (e.g., cross-level interactions; Gignac & Szodorai, 2016) and 0.05 for the within-person associations (Adachi & Willoughby, 2015).

### Data availability

All materials of the current study, including the preregistration (https://osf.io/zgr2k/) and the syntaxes in Mplus and R (https://osf.io/whnmd/), and preregistered materials of the larger project including the sampling plan, data collection procedure, and sample size rationale (https://osf.io/327cx), are publicly available on the Open Science Framework (OSF). The anonymized data set that was used for the current study is available in Figshare (see 10.21942/uva.16929505.v2).

## Results

### Descriptives and correlations

As shown in Table 1, adolescents spent on average 15 min (*SD* = 15.8) per hour on social media and experienced on average little distraction (*M* = 1.84, *SD* = 1.81). Both the between-person (r = 0.47) and within-person correlations (r = 0.18) of SMU with distraction were significantly positive. The intra-class correlations (ICCs) of SMU and distraction were both 0.51, indicating that practically half of the variance in SMU and distraction can be attributed to changes within persons, and half of the variance can be attributed to differences between persons.

**Table 1:** Descriptive statistics and correlations for all study variables

Study variable	Descriptives			Correlations				
	Range	*M*	*SD*	1	2	3	4	5
1. Distraction	0–6	1.84	1.81	—	.18^***^			
2. SMU	0–60	14.46	15.83	.47^***^	—			
3. FoMO	1–5	2.43	1.02	.12^***^	.11^***^	—		
4. FAE	1–5	3.06	1.06	.10^***^	.08^***^	.17^***^	—	
5. SCS	1–5	2.17	0.86	–.07^***^	–.05^***^	.01	.02^*^	—
6. PR	1–5	2.04	0.74	.08^***^	.00	.15^***^	.08^***^	.21^***^

*Note. SMU* social media use, *FoMO* fear of missing out, *FAE* friendship accessibility expectations, *SCS* self-control strategies, *PR* parental restrictions. The correlation above the diagonal line represents the within-person correlation and the correlations below the diagonal line represent between-person correlations. Because FoMO, FAE, SCS, and PR were measured only once, no within-person associations are available. ^*^ *p* < .05 ^**^ *p* < .01 ^***^ *p* < .001

### Between-person, within-person, and person-specific associations

**Fig. 1:**
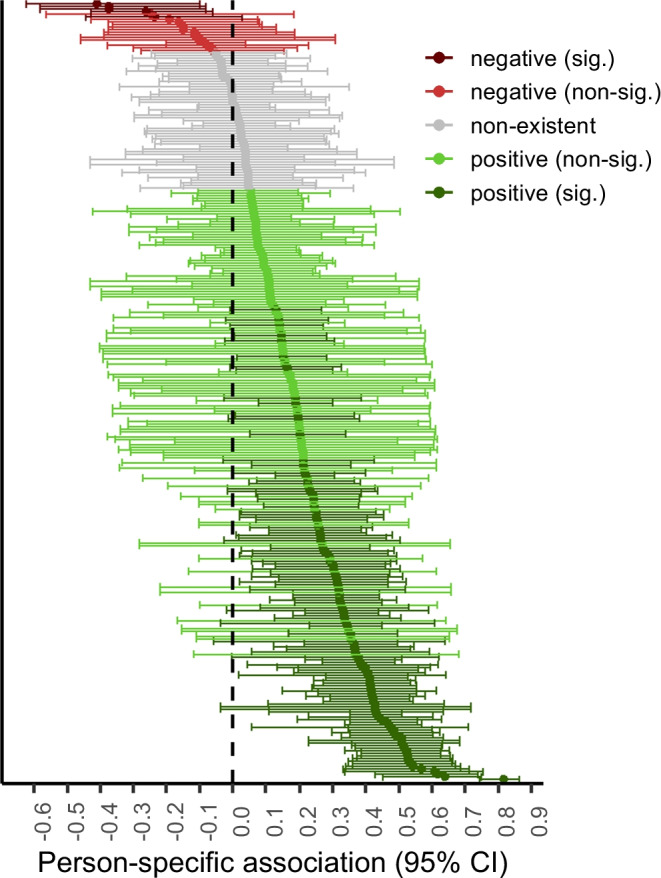
Distribution of the person-specific associations of SMU with distraction and person-specific credible intervals. *Note.* Each shaded point represents a person-specific association. Each bar represents the person-specific Bayesian credible interval. Dark red, light red, grey, light green, and dark green points indicate significant negative (2%), non-significant negative (5%), non-existent (18%), non-significant positive (38%), and significant positive associations (38%), respectively

We first examined the between-person, average within-person, and person-specific associations of SMU with distraction. Model 1 (see Table 2) showed a positive between-person (β = 0.48, *p* < 0.001) and a positive within-person association of SMU with distraction (β = 0.18, *p* < 0.001). This indicates that on average adolescents who spent more time using social media than their peers also experienced more distraction than their peers, and that on average adolescents experienced more distraction at moments when they spent more time using social media. The person-specific associations of SMU with distraction ranged from β = -0.41 to β =  +0.82. Based on our SESOI, the association of SMU with distraction was negative for 7% of adolescents (β < -0.05, *n* = 21), non-existent to very small for 16% of adolescents (-0.05 ≤ β ≤ 0.05, *n* = 49), and positive for 77% of adolescents (β > 0.05, *n* = 230). Based on person-specific significance tests, 38% were significantly positive, 2% significantly negative, and 61% not significant. Figure 1 shows the distribution of all person-specific associations and credible intervals around these person-specific associations.

**Table 2:** Model parameters of the fixed and random effects models

	Model 1			Model 2		
	*b*	β	95% CI	*b*	β	95% CI
Fixed effects						
*Within-person*						
Time ➔ Distraction	0.044	.057^***^	[ .044, .069]	0.044	.057^***^	[ .044, .070]
Day of the week ➔ Distraction	0.011	.008	[–.021, .038]	0.013	.010	[–.020, .038]
SMU ➔ Distraction (Beta)	0.022	.183^***^	[ .163, .201]	0.022	.185^***^	[ .166, .204]
*Between-person*						
SMU ➔ Distraction	0.053	.476^***^	[ .381, .563]	0.045	.399^***^	[ .295, .497]
FoMO ➔ Distraction				0.174	.138^**^	[ .028, .243]
FAE ➔ Distraction				0.187	.153^**^	[ .042, .263]
SCS ➔ Distraction				–0.148	–.097^*^	[–.203, .009]
PR ➔ Distraction				0.156	.090	[-.018, .201]
FoMO ➔ SMU				2.328	.206^**^	[ .080, .328]
FAE ➔ SMU				1.923	.176^**^	[ .054, .294]
SCS ➔ SMU				–0.571	–.042	[–.164, .079]
PR ➔ SMU				0.497	.032	[–.092, .152]
FoMO ➔ Beta (H1)				0.001	.022	[–.131, .170]
FAE ➔ Beta (H2)				–0.003	–.127	[–.276, .028]
SCS ➔ Beta (H3)				0.000	–.005	[-.157, .152]
PR ➔ Beta (H4)				0.003	.081	[-.077, .233]
Random effect	σ^2^		95% CI	σ^2^		95% CI
SMU ➔ Distraction	0.001^***^		[0.001, 0.001]	0.001^***^		[0.001, 0.001]
Other variances (residual)	σ^2^		95% CI	σ^2^		95% CI
Distraction (within-person)	1.479^***^		[1.452, 1.508]	1.479^***^		[1.452, 1.508]
Distraction (between-person)	1.301^***^		[1.109, 1.549]	1.207^***^		[1.022, 1.445]
Explained variance	R^2^		95% CI	R^2^		95% CI
*Within-person*						
Distraction	.088^***^		[.081, .097]	.089^***^		[.080, .097]
*Between-person*						
Distraction	.226^***^		[.145, .317]	.304^***^		[.216, .397]
SMU				.107^***^		[.046, .185]
Beta				.039^***^		[.006, .106]

*Note. b*s are unstandardized effects. βs are standardized effects using STDY for the categorical and STDYX for the continuous variables. The day of the week is coded as 0 for weekdays and 1 for weekend days. *SMU* social media use, *FoMO* fear of missing out, *FAE* friendship accessibility expectations, *SCS* self-control strategies, *PR* parental restrictions. *p* values represent one-tailed Bayesian *p* values (McNeish & Hamaker, 2020). ^*^ *p* < .05 ^**^ *p* < .01 ^***^ *p* < .001

**Fig. 2:**
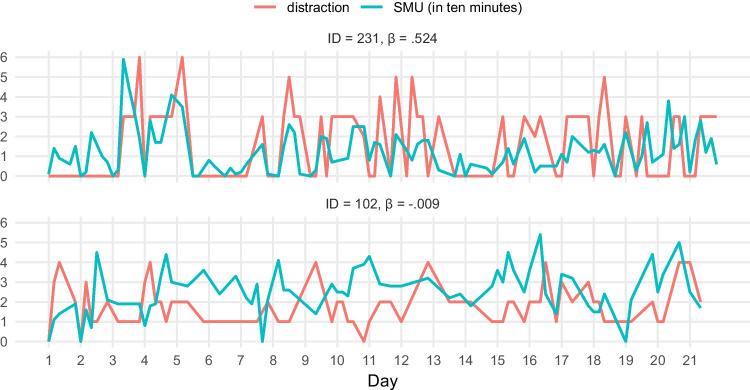
*N* = 1 time series showing the person-specific association of SMU with distraction. *Note.* The x-axis shows the day of the study (range 1–21). The y-axis shows the value of distraction (red; 0 = *not at all* to 6 = *completely*) and SMU in 10 min (blue-green; 0 = *0 min* to 6 = *60 min*). The upper graph represents the time series of a participant for whom the association of SMU with distraction was strongly positive, and the lower graph represents the time series of a participant for whom the association was non-existent

To illustrate the difference in person-specific associations, Fig. 2 includes two *N* = 1 time series of the co-fluctuation between SMU and distraction for one participant with a positive person-specific association (upper time series) and one with a non-existent person-specific association (lower time series). For the participant with a positive association, SMU and distraction co-fluctuated regularly, meaning that increases in SMU were often accompanied by increases in distraction, and decreases in SMU were often accompanied by decreases in distraction. For the participant for whom there was no association between SMU and distraction, increases in SMU were accompanied by increases in distraction at some moments and by decreases in distraction at other moments.

In addition to the associations between SMU and distraction, we also explored the associations between the social connectivity and disconnectivity factors. At the between-person level, FoMO (β = 0.14, *p* < 0.01) and friendship accessibility expectations (β = 0.15, *p* < 0.01) were positively associated with distraction, whereas self-control strategies was negatively associated with distraction (β = -0.10, *p* < 0.05; see Model 2 in Table 2). This indicates that adolescents with higher levels of FoMO and friendship accessibility expectations, and fewer self-control strategies than their peers had a higher tendency to get distracted than their peers. In addition, FoMO (β = 0.21, *p* < 0.01) and friendship accessibility expectations (β = 0.18, *p* < 0.01) were positively associated with SMU, implying that adolescents with higher levels of FoMO and friendship accessibility expectations than their peers generally spent more time on social media than their peers. Parental restrictions were not related to adolescents’ level of distraction or SMU.

### Investigating hypotheses

**Fig. 3:**
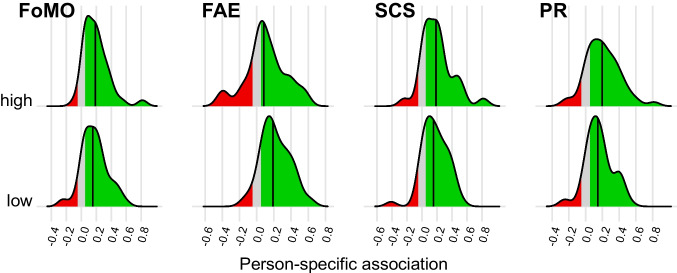
Distribution of the person-specific associations of SMU with distraction for adolescents with high and low levels of FoMO, friendship accessibility expectations (FAE), self-control strategies (SCS), and parental restrictions (PR). *Note.* The black solid vertical lines indicate the overall within-person associations of SMU and distraction for each level of the moderator. Negative, non-existent, and positive person-specific associations are presented in red, grey, and green, respectively

Our preregistered hypotheses predicted that adolescents who had higher levels of FoMO (H1), higher friendship accessibility expectations (H2), who used self-control strategies less frequently (H3), and who had fewer parental restrictions (H4) would have more social media-related distraction than their peers. However, none of the hypotheses were confirmed (see Model 2). We found no evidence that FoMO (β = 0.02, *p* = 0.39), friendship accessibility expectations (β = -0.13, *p* = 0.06), self-control strategies (β = 0.00, *p* = 0.48), or parental restrictions (β = 0.08, *p* = 0.16) accounted for the differences in person-specific associations of SMU with distraction. Accordingly, Fig. 3 shows that the distribution of the person-specific associations does not differ for adolescents high and low in FoMO, friendship accessibility expectations, self-control strategies, and parental restrictions.

## Discussion

Social media-related distraction is a major concern among parents, teachers, and the society at large, especially during the COVID-19 pandemic, when adolescents’ screen time was at an all-time high (Nagata et al., 2021; Paschke et al., 2021). The current study showed that social media-related distraction is indeed common among adolescents, with 77% of adolescents experiencing more distraction as they spend more time using social media. We partly replicated a recent ESM study by Siebers et al. (2021), which was conducted in November 2019 among the same sample of adolescents, six months before the current study. In comparison to Siebers et al. (2021), we found both a stronger positive between-person association (β = 0.48 vs β = 0.31 in Siebers et al.) and a stronger within-person association of SMU with distraction (β = 0.18 vs β = 0.12 in Siebers et al.).

Our findings imply that across only six months, both the between- and within-person associations of SMU with distraction have increased. Whereas Siebers et al. (2021) found that SMU explained 10% of the between-person variance in distraction, in the current study it explained 23% of this variance. An explanation for these increases in between- and with-person associations may be that the study of Siebers et al. was conducted just before the COVID-19 pandemic, while the current study took place in June 2020, amid the pandemic, when smartphones and social media were more popular than before (Nagata et al., 2021; Paschke et al., 2021) and had more potential than ever to distract adolescents’ attention.

In line with Siebers et al. (2021), we found that the majority of adolescents experienced social media-related distraction (77% vs 83% in Siebers et al.). This preponderance has also been reported in related work, in which 93% of students experienced smartphone-induced procrastination (Aalbers et al., 2021). However, when comparing the results of the distraction and procrastination studies with those of studies on well-being (Beyens et al., 2020; Valkenburg et al., 2022) and depression (Rodriguez et al., 2021), it becomes clear that the distribution of the person-specific associations in the distraction and procrastination studies is far more skewed to the right than the distribution of the person-specific associations in the well-being and depression studies, which are clustered around zero. This means that the associations of SMU with distraction and procrastination are considerably more sizeable than those of SMU with well-being and depression.

Even though our results showed that most adolescents experienced social media-related distraction, some adolescents did not. Relying on Self-Determination Theory (SDT; Ryan & Deci, 2000), we tried to explain these differences by examining whether two connectivity factors and two disconnectivity factors accounted for differences in the person-specific associations. More specifically, we hypothesized that the differences in social media-related distraction could be explained by differences in FoMO (H1), friendship accessibility expectations (H2), adolescents’ self-control strategies (H3), and parental restrictions (H4). However, none of these hypotheses received support.

A potentially convincing explanation why the social connectivity factors could not explain the heterogeneity in the person-specific associations may be that social media-related distraction is not a consequence of adolescents’ dispositions, but of the addictive design of social media platforms (Vanden Abeele et al., 2022). From this perspective, SMU is considered to be impulse-response behavior, over which users have little conscious control (Vanden Abeele, 2020). To avoid social media-related distractions, adolescents may adopt self-control strategies, such as making sure that their phone is not around or turning on airplane mode (Brevers & Turel, 2019). And parents may set limits on when and how long adolescents can use social media (Shin & Li, 2016; van den Eijnden et al., 2021). However, this addictive design explanation does not seem plausible, as we found that the degree of social media-related distraction that adolescents experienced did not depend on how often adolescents adopted social media self-control strategies or to what extent parents imposed phone restrictions.

A more convincing explanation is that social media-related distraction depends on adolescents’ capacity to manage situational temptations (Vanden Abeele et al., 2022). Media effects theories, such as the Differential Susceptibility to Media Effects Model (Valkenburg & Peter, 2013), argue that differences in adolescents’ susceptibility to media effects cannot only be explained by stable dispositions but also by transient dispositions. For example, adolescents may temporarily fail to focus their attention as a result of increased depletion (Englert & Bertrams, 2015; Garrison et al., 2019), boredom (Hunter & Eastwood, 2018), or sleep deprivation (Xanidis & Brignell, 2016). This explanation seems plausible, as Fig. 1 shows that many person-specific associations have a relatively wide credible interval. This indicates that there is considerable fluctuation in the association between SMU and distraction within a person. This is also illustrated by the *N* = 1 time series in Fig. 2: An increase in adolescents’ SMU may be associated with an increase in distraction at some moments but not at other moments. For example, the upper *N* = 1 time series reflects a strong positive association of SMU with distraction, which suggests predominantly positive momentary associations within this adolescent. However, the lower *N* = 1 time series reflects an overall null effect, which is comprised of momentary positive and negative associations of SMU with well-being that cancel each other out. Thus, the presence of social media-related distraction evidently varies within adolescents and may depend on person- and context-specific factors (Vanden Abeele et al., 2022). Therefore, future research is encouraged to investigate to what extent differences in social media-related distraction can be explained by such factors.

Even though the social connectivity and disconnectivity factors could not explain the heterogeneity in social media-related distraction, we believe that both deserve further research attention. Specifically, even though FoMO and friendship accessibility expectations did not moderate the within-person association of SMU and distraction, both factors were significantly positively associated with adolescents’ SMU. This confirms what we would expect based on SDT and previous research (Allen et al., 2014; Beyens et al., 2016): Adolescents with a stronger need for social connectivity than their peers spent more time using social media than their peers. Moreover, adolescents who experienced more FoMO, who had higher friendship accessibility expectations, or who less often used self-control strategies than their peers experienced more distraction than their peers.

While the current study found that SMU often coincides with distraction among adolescents, an open question remains whether the social media-related distractions uncovered in the current study are the result of adolescents’ SMU or their more general problematic smartphone use. Whereas social media can be accessed on multiple devices, the smartphone is by far the most popular device for using social media (Bayer et al., 2022; Deng et al., 2019). As such, it may not be social media but the smartphone that is causing distraction. For instance, social media notifications frequently manifest via smartphones, causing adolescents to create smartphone checking routines to see whether they received new messages from others. Consequently, adolescents may develop habitual smartphone use (Bayer & LaRose, 2018). Such habitual use may even be more distracting than social media in itself, as it occurs frequently and fragmentedly (Heitmayer & Lahlou, 2020; Oulasvirta et al., 2012). Thus, future research is encouraged to investigate to what extent social media-related distraction reflects smartphone-related distraction.

Finally, research is needed to investigate how social media-related distraction translates to general failures in self-regulation. Self-regulation consists of multiple aspects, including attentional control (Diamond, 2013; Diehl et al., 2006; Tavares & Freire, 2016) and self-control (Diamond, 2013; Inzlicht et al., 2021). While the findings of our study, along with the findings of previous studies (Aalbers et al., 2021), provide insights into the associations of SMU with different aspects of self-regulation, that is, attentional control and self-control, research is needed that investigates the impact of SMU on adolescents’ general self-regulation.

### Conclusion

Developing attentional control, the ability to focus attention and control potential distractions (Diamond, 2013), is an important task in adolescence (Luna, 2009; Luna et al., 2004). However, the distracting potential of social media may jeopardize this developmental task, as the current study found that many adolescents experienced social media-related distraction. To our knowledge, no existing (social) media effects study has found such sizeable associations and with such a degree of consistency as the current study did. While theoretically valuable, these findings also provide practical implications for practitioners who seek to help adolescents to cope with social media-related distraction. For example, the person-specific associations uncovered in the current study may help practitioners to develop tailored prevention and intervention programs and provide personalized advice to strengthen adolescents’ capacity to manage social media temptations. The fact that the (dis)connectivity factors could not explain why many adolescents experience social media-related distraction, while some others do not, seems to confirm what parents and educators have been saying for years: Social media are a powerful distractor for many adolescents.

## References

[CR1] Aalbers G, McNally RJ, Heeren A, de Wit S, Fried EI (2019). Social media and depression symptoms: A network perspective. Journal of Experimental Psychology.

[CR2] Aalbers, G., vanden Abeele, M. M., Hendrickson, A. T., de Marez, L., & Keijsers, L. (2021). Caught in the moment: Are there person-specific associations between momentary procrastination and passively measured smartphone use? *Mobile Media & Communication, 10*(1), 115–135. 10.1177/2050157921993896

[CR3] Adachi P, Willoughby T (2015). Interpreting effect sizes when controlling for stability effects in longitudinal autoregressive models: Implications for psychological science. European Journal of Developmental Psychology.

[CR4] Al-Furaih SA, Al-Awidi HM (2021). Fear of missing out (FoMO) among undergraduate students in relation to attention distraction and learning disengagement in lectures. Education and Information Technologies.

[CR5] Allen, K. A., Ryan, T., Gray, D. L., McInerney, D. M., & Waters, L. (2014). Social media use and social connectedness in adolescents: The positives and the potential pitfalls. *Australian Educational Developmental Psychologist,**31*(1), 18–31. 10.1017/edp.2014.2

[CR6] Barry CT, Wong MY (2020). Fear of missing out (FoMO): A generational phenomenon or an individual difference?. Journal of Social and Personal Relationships.

[CR7] Bayer, J. B., Anderson, I. A., & Tokunaga, R. (2022). Building and breaking social media habits. *Current Opinion in Psychology*. 10.1016/j.copsyc.2022.101303 10.1016/j.copsyc.2022.101303 35255413

[CR8] Bayer JB, Campbell SW, Ling R (2016). Connection cues: Activating the norms and habits of social connectedness. Communication Theory.

[CR9] Bayer, J. B., & LaRose, R. (2018). Technology habits: Progress, problems, and prospects. In B. Verplanken (Ed.), *The psychology of habit: Theory, mechanisms, change, and contexts* (pp. 111–130). Springer. 10.1007/978-3-319-97529-0_7

[CR10] Beyens I, Frison E, Eggermont S (2016). “I don’t want to miss a thing”: Adolescents’ fear of missing out and its relationship to adolescents’ social needs, Facebook use, and Facebook related stress. Computers in Human Behavior.

[CR11] Beyens I, Pouwels JL, van Driel II, Keijsers L, Valkenburg PM (2020). The effect of social media on well-being differs from adolescent to adolescent. Scientific Reports.

[CR12] Beyens I, Valkenburg PM (2019). Parental media mediation in adolescence: A comparative study of parent and adolescent reports. Journal of Broadcasting & Electronic Media.

[CR13] Brevers D, Turel O (2019). Strategies for self-controlling social media use: Classification and role in preventing social media addiction symptoms. Journal of Behavioral Addictions.

[CR14] Brooks S (2015). Does personal social media usage affect efficiency and well-being?. Computers in Human Behavior.

[CR15] Casey BJ, Caudle K (2013). The teenage brain: Self control. Current Directions in Psychological Science.

[CR16] Chang, W., Cheng, J., Allaire, J., Sievert, C., Schloerke, B., Xie, Y., Allen, J., McPherson, J., Dipert, A., & Borges, B. (2020). *shiny: Web Application Framework for R. R package version 1.4.0.2.* https://CRAN.R-project.org/package=shiny. Accessed 1 Nov 2019.

[CR17] Chen, Y., Li, R., & Liu, X. (2021). Relatedness frustration and compensatory behaviors in social networking sites among Chinese college students: Role of self-control failure. *Current Psychology*. 10.1007/s12144-021-01440-0

[CR18] Chin, B., Lindsay, E. K., Greco, C. M., Brown, K. W., Smyth, J. M., Wright, A. G., & Creswell, J. D. (2020). Mindfulness interventions improve momentary and trait measures of attentional control: Evidence from a randomized controlled trial. *Journal of experimental psychology: General*. 10.1037/xge0000969 10.1037/xge0000969 PMC9245911 32969686

[CR19] Chotpitayasunondh V, Douglas KM (2016). How “phubbing” becomes the norm: The antecedents and consequences of snubbing via smartphone. Computers in Human Behavior.

[CR20] Deng T, Kanthawala S, Meng J, Peng W, Kononova A, Hao Q, Zhang Q, David P (2019). Measuring smartphone usage and task switching with log tracking and self-reports. Mobile Media & Communication.

[CR21] Diamond A (2013). Executive functions. Annual Review of Psychology.

[CR22] Diehl M, Semegon AB, Schwarzer R (2006). Assessing attention control in goal pursuit: A component of dispositional self-regulation. Journal of Personality Assessment.

[CR23] Dontre AJ (2021). The influence of technology on academic distraction: A review. Human Behavior and Emerging Technologies.

[CR24] Duckworth AL., Gendler TS, Gross JJ (2016). Situational strategies for self-control. Perspectives on Psychological Science.

[CR25] Duckworth AL, Milkman KL, Laibson D (2018). Beyond willpower: Strategies for reducing failures of self-control. Psychological Science in the Public Interest.

[CR26] Englert C, Bertrams A (2015). Integrating attentional control theory and the strength model of self-control. Frontiers in Psychology.

[CR27] Franchina V, Vanden Abeele M, van Rooij AJ, Lo Coco G, De Marez L (2018). Fear of missing out as a predictor of problematic social media use and phubbing behavior among Flemish adolescents. International Journal of Environmental Research and Public Health.

[CR28] Garrison KE, Finley AJ, Schmeichel BJ (2019). Ego depletion reduces attention control: Evidence from two high-powered preregistered experiments. Personality and Social Psychology Bulletin.

[CR29] Gignac GE, Szodorai ET (2016). 2016/11/01/). Effect size guidelines for individual differences researchers. Personality and Individual Differences.

[CR30] Grolnick WS, Deci EL, Ryan RM, Grusec JE, Kuczynski L (1997). Internalization within the family: The self-determination theory perspective. Parenting and children's internalization of values: A handbook of contemporary theory.

[CR31] Hall JA, Baym NK (2012). Calling and texting (too much): Mobile maintenance expectations,(over) dependence, entrapment, and friendship satisfaction. New Media & Society.

[CR32] Heitmayer, M., & Lahlou, S. (2020). Why are smartphones disruptive? An empirical study of smartphone use in real-life contexts. *Computers in Human Behavior*,* 116*, 1–12 10.1016/j.chb.2020.106637

[CR33] Hunter A, Eastwood JD (2018). Does state boredom cause failures of attention? Examining the relations between trait boredom, state boredom, and sustained attention. Experimental Brain Research.

[CR34] Inzlicht M, Werner KM, Briskin JL, Roberts BW (2021). Integrating models of self-regulation. Annual Review of Psychology.

[CR35] Johannes, N., Veling, H., Verwijmeren, T., & Buijzen, M. (2019). Hard to resist? The effect of smartphone visibility and notifications on response inhibition. *Journal of Media Psychology,**31*(4), 214. 10.1027/1864-1105/a000248

[CR36] Johannes, N., Meier, A., Reinecke, L., Ehlert, S., Setiawan, D. N., Walasek, N., Dienlin, T., Buijzen, M., & Veling, H. (2020). The relationship between online vigilance and affective well-being in everyday life: Combining smartphone logging with experience sampling. *Media Psychology, 24*(5), 581–605. 10.1080/15213269.2020.1768122

[CR37] Jong ST, Drummond MJN (2016). Hurry up and ‘like’ me: immediate feedback on social networking sites and the impact on adolescent girls. Asia-Pacific Journal of Health, Sport and Physical Education.

[CR38] Lakens D, Scheel AM, Isager PM (2018). Equivalence testing for psychological research: A tutorial. Advances in Methods and Practices in Psychological Science.

[CR39] Levine LE, Waite BM, Bowman LL (2007). Electronic media use, reading, and academic distractibility in college youth. CyberPsychology & Behavior.

[CR40] Luna, B. (2009). Developmental changes in cognitive control through adolescence. *Advances in Child Development and Behavior,**37*, 233–278. 10.1016/S0065-2407(09)03706-9 10.1016/s0065-2407(09)03706-9 PMC2782527 19673164

[CR41] Luna, B., Garver, K. E., Urban, T. A., Lazar, N. A., & Sweeney, J. A. (2004). Maturation of cognitive processes from late childhood to adulthood. *Child Development,**75*(5), 1357–1372. 10.1111/j.1467-8624.2004.00745.x 10.1111/j.1467-8624.2004.00745.x 15369519

[CR42] Marino, C., Gini, G., Angelini, F., Vieno, A., & Spada, M. (2020). Social norms and e-motions in problematic social media use among adolescents. *Addictive Behaviors Reports, 11*, 100250. 10.1016/j.abrep.2020.100250 10.1016/j.abrep.2020.100250 PMC7244919 32467839

[CR43] McNeish D, Hamaker EL (2020). A primer on two-level dynamic structural equation models for intensive longitudinal data in Mplus. Psychological Methods.

[CR44] Milyavskaya M, Saffran M, Hope N, Koestner R (2018). Fear of missing out: Prevalence, dynamics, and consequences of experiencing FOMO. Motivation and Emotion.

[CR45] Muthén LK, Muthén BO (2017). Mplus user's guide.

[CR46] Nagata, J. M., Cortez, C. A., Cattle, C. J., Ganson, K. T., Iyer, P., Bibbins-Domingo, K., & Baker, F. C. (2021). Screen time use among US adolescents during the COVID-19 pandemic: Findings from the Adolescent Brain Cognitive Development (ABCD) study. *Jama Pediatrics, 176(1), 94–96*. 10.1001/jamapediatrics.2021.4334 10.1001/jamapediatrics.2021.4334 PMC8561427 34724543

[CR47] Nesi J, Choukas-Bradley S, Prinstein MJ (2018). Transformation of adolescent peer relations in the social media context: Part 1—A theoretical framework and application to dyadic peer relationships. Clinical Child and Family Psychology Review.

[CR48] Orkibi, H., & Ronen, T. (2017). Basic psychological needs satisfaction mediates the association between self-control skills and subjective well-being. *Frontiers in Psychology, 8*(JUN), 1–10. 10.3389/fpsyg.2017.00936 10.3389/fpsyg.2017.00936 PMC5461363 28638362

[CR49] Oulasvirta A, Rattenbury T, Ma L, Raita E (2012). Habits make smartphone use more pervasive. Personal and Ubiquitous Computing.

[CR50] Paschke, K., Austermann, M. I., Simon-Kutscher, K., & Thomasius, R. (2021). Adolescent gaming and social media usage before and during the COVID-19 pandemic. *Sucht, 67*(1), 13–22*.*10.1024/0939-5911/a000694

[CR51] Perrone, M. P. (2016). *#FoMO: Establishing validity of the Fear of Missing Out Scale with an adolescent population* [Doctoral Dissertation]. School Psychology Alfred. http://hdl.handle.net/10829/7399. Accessed 8 Oct 2020.

[CR52] Przybylski AK, Murayama K, DeHaan CR, Gladwell V (2013). Motivational, emotional, and behavioral correlates of fear of missing out. Computers in Human Behavior.

[CR53] Rideout V, Robb MB (2018). Social media, social life: Teens reveal their experiences.

[CR54] Rodriguez, M., Aalbers, G., & McNally, R. J. (2021). Idiographic network models of social media use and depression symptoms. *Cognitive Therapy and Research, 46*, 124–132. 10.1007/s10608-021-10236-2

[CR55] Rosen LD, Carrier LM, Cheever NA (2013). Facebook and texting made me do it: Media-induced task-switching while studying. Computers in Human Behavior.

[CR56] Rozgonjuk D, Elhai JD, Ryan T, Scott GG (2019). Fear of missing out is associated with disrupted activities from receiving smartphone notifications and surface learning in college students. Computers & Education.

[CR57] Ryan RM, Deci EL (2000). Self-determination theory and the facilitation of intrinsic motivation, social development, and well-being. American Psychologist.

[CR58] Schneider, F. M., & Hitzfeld, S. (2021). I ought to put down that phone but I phub nevertheless: Examining the predictors of phubbing behavior. *Social Science Computer Review, 39*(6), 1075–1088. 10.1177/0894439319882365

[CR59] Schultzberg M, Muthén B (2018). Number of subjects and time points needed for multilevel time-series analysis: A simulation study of dynamic structural equation modeling. Structural Equation Modeling: A Multidisciplinary Journal.

[CR60] Schuurman, N. K., Ferrer, E., de Boer-Sonnenschein, M., & Hamaker, E. L. (2016). How to compare cross-lagged associations in a multilevel autoregressive model. *Psychological Methods,**21*(2), 206–221. 10.1037/met0000062 10.1037/met0000062 27045851

[CR61] Sheldon, K. M., Abad, N., & Hinsch, C. (2011). A two-process view of Facebook use and relatedness need-satisfaction: Disconnection drives use, and connection rewards it. *Journal of Personality and Social Psychology, 100*(4), 766–775. 10.1037/a0022407 10.1037/a0022407 21280967

[CR62] Shin W, Li B (2016). Parental mediation of children’s digital technology use in Singapore. Journal of Children and Media.

[CR63] Siebers, T., Beyens, I., Pouwels, J. L., & Valkenburg, P. M. (2021). Social media and distraction: An experience sampling study among adolescents. *Media Psychology, 25*(3), 343–366. 10.1080/15213269.2021.1959350

[CR64] Statistics Netherlands. (2020). *Kerncijfers wijken en buurten 2020 [StatLine]*. https://www.cbs.nl/nl-nl/maatwerk/2020/29/kerncijfers-wijken-en-buurten-2020. Accessed 14 April 2022.

[CR65] Tavares, D., & Freire, T. (2016). Flow experience, attentional control, and emotion regulation: Contributions for a positive development in adolescents. *Psicologia, 30*(2), 77–94. 10.17575/rpsicol.v30i2.1119

[CR66] Valkenburg, P. M., Beyens, I., Pouwels, J. L., van Driel, I. I., & Keijsers, L. (2022). Social media browsing and adolescent well-being: Challenging the “Passive Social Media Use Hypothesis”. *Journal of Computer-Mediated Communication, 27*(1), 1–19. 10.1093/jcmc/zmab015

[CR67] Valkenburg PM, Peter J (2013). The differential susceptibility to media effects model. Journal of Communication.

[CR68] Valkenburg PM, Piotrowski JT, Hermanns J, de Leeuw R (2013). Developing and validating the perceived parental media mediation scale: A self-determination perspective. Human Communication Research.

[CR69] van den Eijnden RJJM, Geurts SM, Ter Bogt TFM, van der Rijst VG, Koning IM (2021). Social media use and adolescents’ sleep: A longitudinal study on the protective role of parental rules regarding Internet use before sleep. International Journal of Environmental Research and Public Health.

[CR70] Vanden Abeele, M. M. P. (2020). Digital wellbeing as a dynamic construct. *Communication Theory, 31*(4), 932–955. 10.1093/ct/qtaa024

[CR71] Vanden Abeele, M. M. P., Halfmann, A., & Lee, E. W. J. (2022). Drug, demon, or donut? Theorizing the relationship between social media use, digital well-being and digital disconnection. *Current Opinion in Psychology,**45*, 101295. 10.1016/j.copsyc.2021.12.007 10.1016/j.copsyc.2021.12.007 35123383

[CR72] van Driel, I. I., Pouwels, J. L., Beyens, I., Keijsers, L., & Valkenburg, P. M. (2019). *‘Posten, scrollen, appen en snappen’: Jongeren (14–15 jaar) en social media in 2019*. Center for Research on Children, Adolescents, and the Media (CcaM).

[CR73] Wang LP, Maxwell SE (2015). On disaggregating between-person and within-person effects with longitudinal data using multilevel models. Psychological methods.

[CR74] Widhiarso, W. (2010). Estimate reliability measurement for multidimensional scales. *SSRN Electronic Journal.* 10.2139/ssrn.1597532

[CR75] Willems, Y., Boesen, N., Li, J., Finkenauer, C., & Bartels, M. (2019). The heritability of self-control: A meta-analysis. *Neuroscience & Biobehavioral Reviews, 100*(February), 324–334. 10.1016/j.neubiorev.2019.02.012 10.1016/j.neubiorev.2019.02.012 30822436

[CR76] Xanidis N, Brignell CM (2016). The association between the use of social network sites, sleep quality and cognitive function during the day. Computers in Human Behavior.

[CR77] Xie J-Q, Rost DH, Wang F-X, Wang J-L, Monk RL (2021). The association between excessive social media use and distraction: An eye movement tracking study. Information & Management.

